# Relationships between functional alpha and beta diversities of flea parasites and their small mammalian hosts

**DOI:** 10.1017/S0031182024000283

**Published:** 2024-04

**Authors:** Boris R. Krasnov, Irina S. Khokhlova, M. Fernanda López Berrizbeitia, Sonja Matthee, Juliana P. Sanchez, Georgy I. Shenbrot, Luther van der Mescht

**Affiliations:** 1 Mitrani Department of Desert Ecology, Swiss Institute for Dryland Environmental and Energy Research, Jacob Blaustein Institutes for Desert Research, Ben-Gurion University of the Negev, Sede Boqer Campus, 8499000 Midreshet Ben-Gurion, Israel; 2 French Associates Institute for Agriculture and Biotechnology of Drylands, Jacob Blaustein Institutes for Desert Research, Ben-Gurion University of the Negev, Sede Boqer Campus, 8499000 Midreshet Ben-Gurion, Israel; 3 Programa de Conservación de los Murciélagos de Argentina (PCMA) and Instituto de Investigaciones de Biodiversidad Argentina (PIDBA)-CCT CONICET Noa Sur (Consejo Nacional de Investigaciones Científicas y Técnicas), Facultad de Ciencias Naturales e IML, UNT, and Fundación Miguel Lillo, Miguel Lillo 251, 4000 San Miguel de Tucumán, Argentina; 4 Stellenbosch University, Private Bag X1, Matieland 7602, South Africa; 5 Centro de Investigaciones y Transferencia del Noroeste de la Provincia de Buenos Aires – CITNOBA (CONICET-UNNOBA), Ruta Provincial 32 Km 3.5, 2700 Pergamino, Argentina; 6 Clinvet International (Pty) Ltd, Universitas, Uitsig Road, Bloemfontein 9338, South Africa; 7 Department of Zoology and Entomology, University of the Free State, 205 Nelson Mandela Dr, Park West, Bloemfontein 9301, South Africa.

**Keywords:** biogeographic realms, fleas, functional alpha diversity, functional alpha diversity components, functional beta diversity, mammals

## Abstract

We studied the relationships between functional alpha and beta diversities of fleas and their small mammalian hosts in 4 biogeographic realms (the Afrotropics, the Nearctic, the Neotropics and the Palearctic), considering 3 components of alpha diversity (functional richness, divergence and regularity). We asked whether (a) flea alpha and beta diversities are driven by host alpha and beta diversities; (b) the variation in the off-host environment affects variation in flea alpha and beta diversities; and (c) the pattern of the relationship between flea and host alpha or beta diversities differs between geographic realms. We analysed alpha diversity using modified phylogenetic generalized least squares and beta diversity using modified phylogenetic generalized dissimilarity modelling. In all realms, flea functional richness and regularity increased with an increase in host functional richness and regularity, respectively, whereas flea functional divergence correlated positively with host functional divergence in the Nearctic only. Environmental effects on the components of flea alpha diversity were found only in the Holarctic realms. Host functional beta diversity was invariantly the best predictor of flea functional beta diversity in all realms, whereas the effects of environmental variables on flea functional beta diversity were much weaker and differed between realms. We conclude that flea functional diversity is mostly driven by host functional diversity, whereas the environmental effects on flea functional diversity vary (a) geographically and (b) between components of functional alpha diversity.

## Graphical abstract

**Figure:**
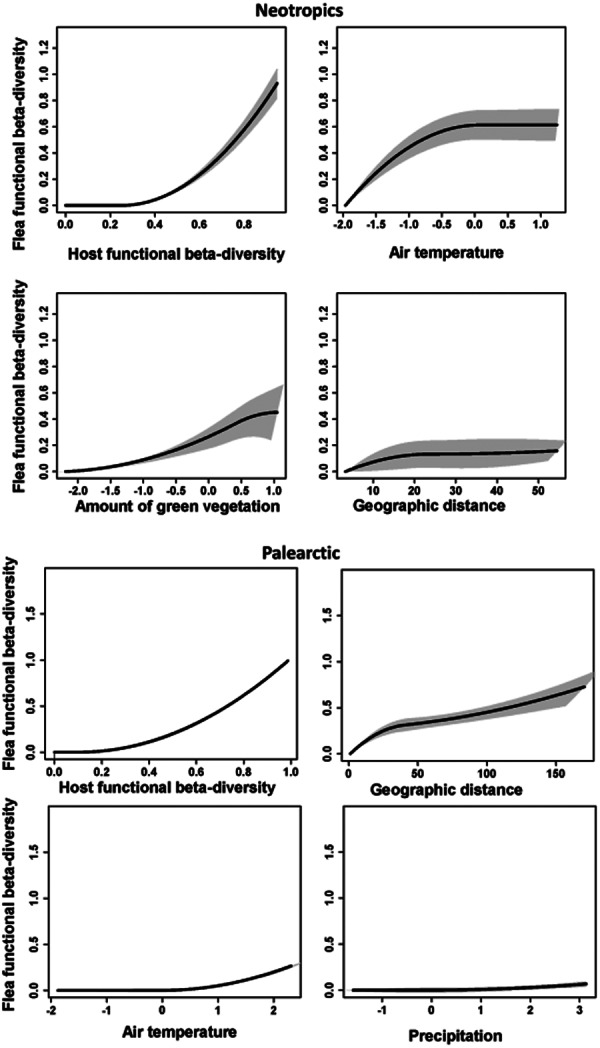

## Introduction

Studying parasite diversity is crucial not only because many parasites are important to medicine and veterinary, but also because parasites, being independently evolved in multiple phylogenetic lineages, present the opportunity for testing various biogeographic and/or evolutionary hypotheses (Poulin and Morand, 2000). Given that parasites ultimately depend on their hosts, it is not surprising that parasite physiology, behaviour, population and community structure, including diversity, are often tightly related to those of their hosts (Krasnov *et al*., 2002; Tschirren *et al*., 2007; Maher and Timm, 2014; Slowinski *et al*., 2018). In other words, parasite traits, phylogeny and ecology are thought to be, to some extent, a product of host traits, phylogeny and ecology, albeit constrained by the evolutionary history of parasites themselves (Poulin, 2021), whereas the effect of environment on, for example, diversity of parasite traits is not always clear. Consequently, geographic variation of the patterns of parasite diversity and relative roles of host diversity and environmental factors on these patterns are far from being completely understood.

Biological diversity is represented not only by species richness and composition (compositional diversity), but also by the richness and composition of phylogenetic lineages (phylogenetic diversity) and functional traits (functional diversity) (Tilman *et al*., 1997; Webb *et al*., 2002; Cavender-Bares *et al*., 2009; De Bello *et al*., 2010; Le Bagousse-Pinguet *et al*., 2019). There is much evidence of the positive relationships between host and parasite diversities, in terms of species richness, for a variety of host and parasite taxa, geographic regions and environments (Krasnov *et al*., 2004; Hechinger and Lafferty, 2005; Kamiya *et al*., 2014). In other words, compositional parasite alpha diversity (i.e. diversity within a site/region; sensu Whittaker, 1960, 1972) was thought to depend strongly on compositional host alpha diversity. However, a positive relationship between compositional parasite–host alpha diversities appeared to be geographically variable and were found in some, but not other, biogeographic realms (Krasnov *et al*., 2007, but see Krasnov *et al*., 2012 for the results produced by a different type of analysis). In contrast, when compositional parasite diversity was measured as species turnover (i.e. diversity between sites/regions = beta diversity; sensu Whittaker, 1960, 1972), the positive relationship between parasite and host compositional beta diversities seemed to be geographically invariant (e.g. Maestri *et al*., 2017; Eriksson *et al*., 2020; Krasnov *et al*., 2020*a*, 2020*b*).

Studies that have dealt with the relationship between phylogenetic diversities of parasites and their hosts also demonstrated that geographic patterns of this relationship differed depending on the measure of diversity considered. Indeed, flea phylogenetic alpha diversity depended on the phylogenetic alpha diversity of their small mammalian hosts in the Palearctic only but not in the Nearctic, Neotropics or Afrotropics (Krasnov *et al*., 2019*a*; see also Krasnov *et al*., 2012), whereas a positive relationship between parasite and host phylogenetic beta diversity (Clark *et al*., 2018) has been observed in 6 biogeographic realms (Krasnov *et al*., 2023*a*).

Summarizing the results of the studies cited above, it can be suggested that geographic variation in the relationship between host and parasite diversities differs between the alpha and beta diversities of either compositional or phylogenetic diversity. In a nutshell, positive relationships between parasite and host compositional and phylogenetic alpha diversities seem to vary geographically, whereas the relationships between parasite and host compositional and phylogenetic beta diversities seem to be geographically invariant.

In contrast to the relationships between parasite and host compositional and phylogenetic diversities, the relationships between their functional diversities are much less known. To the best of our knowledge, the only study that investigated the effect of host functional diversity on the functional diversity of parasites dealt with parasite diversity at the scale of infracommunities (parasite communities harboured by individual hosts) (Krasnov *et al*., 2019*b*), whereas we are not aware of any study that considered this question at the scale of compound parasite communities (parasite communities harboured by host communities). To fill this gap, here we studied the relationships between functional parasite and host alpha and beta diversities in 4 biogeographic realms (the Afrotropics, the Nearctic, the Neotropics and the Palearctic) in the model of fleas and their small mammalian hosts. We predicted predominantly positive relationships between flea and host functional diversities because (a) the association between the traits of consumer species and consumed species is well established for free-living organisms (e.g. Rezende *et al*., 2007); (b) the importance of trait complementarity between parasites and their hosts has been recognized (McQuaid and Britton, 2013); and (c) the association between flea and small mammalian host traits has been proven, at least for the Palearctic (i.e. fleas possessing certain traits exploit hosts possessing certain traits, while hosts with certain traits harbour parasites with certain traits; Krasnov *et al*., 2016). In addition, many hypotheses explaining latitudinal gradients in functional parasite traits state that parasite traits track host traits (Poulin, 2021). Finally, the functional diversity of parasites has been shown to depend on host traits (e.g. Euclydes *et al*., 2021). Consequently, we asked whether (a) functional flea alpha and beta diversities in different regions within a realm are indeed driven by functional host alpha and beta diversities, respectively, (b) the variation in the off-host environment affects variation in flea functional diversity; and (c) the pattern of the relationship between flea and host functional alpha or beta diversities differs between geographic realms.

It is now commonly recognized that functional alpha diversity is a multifaceted concept that can be characterized by a number of components, namely functional richness, functional divergence and functional regularity (Mason *et al*., 2005; Villéger *et al*., 2008; Tucker *et al*., 2017; Mammola *et al*., 2021; Schmera *et al*., 2023). Schmera *et al*. (2023) proposed a concept of so-called functional diversity units (FDUs) which are discrete entities that represent community members (e.g. species) from a functional perspective. The components of functional alpha diversity are separated by the associated questions. In particular, functional richness answers the question about how many FDUs can be distinguished within a community (i.e. how large is a community in relation to functional traits of its members), whereas functional divergence and functional regularity answer the question about how different and how variable, respectively, these FDUs are. The components of functional alpha diversity can be calculated based on the unrooted functional tree constructed from the matrix of trait (dis)similarity between species in a community (Cardoso *et al*., 2022), where functional richness, functional divergence and functional regularity are represented by the sum, the mean and the variance of the branch lengths, respectively (Cardoso *et al*., 2022; Schmera *et al*., 2023). In the framework of this study, we considered these components of functional diversity separately. This is because different components of functional alpha diversity of parasites can respond differently to either host alpha diversity or environmental factors or both and their patterns may vary between biogeographic realms (Schumm *et al*., 2019).

## Materials and methods

### Data on regional distribution of fleas and hosts

Data on the regional distribution of fleas and small mammalian hosts (Didelphimorphia, Macroscelidea, Eulipotyphla, Rodentia and the ochotonid Lagomorpha) were taken from published regional surveys for 15 regions in the Afrotropics, 23 regions in the Nearctic, 17 regions in the Neotropics and 36 regions in the Palearctic (see lists of region, maps and sources of information in Krasnov *et al*., 2022), including in the analyses mammal species on which at least 1 flea species was recorded. Fleas *Xenopsylla cheopis*, *Xenopsylla brasiliensis*, *Nosopsyllus fasciatus* and *Nosopsyllus londiniensis* characteristic for synanthropic ubiquitous rodents as well as these rodent species (*Rattus norvegicus*, *Rattus rattus* and *Mus musculus*) were not included in the analyses.

### Flea and host traits

Data on flea and host traits were taken from our recent study (Krasnov *et al*., 2023*b*). Flea traits included 2 morphological and 4 ecological traits. Morphological traits were (a) the number of sclerotized ctenidia (no ctenidia, only a pronotal ctenidium, both pronotal and genal ctenidia) and (b) body length (ranked as small, medium or large), whereas ecological traits were (a, b) the number and phylogenetic diversity of host species exploited across a flea's geographic range; (c) the latitudinal span of geographic range; and (d) microhabitat preference (preference to spend the most time in a host's hair, its burrow/nest or no clear preference).

Small mammals were characterized by traits presumably affecting the patterns of flea parasitism (e.g. Krasnov *et al*., 2016). These were 2 morphological (average body mass and relative brain mass), 1 geographic (geographic range size) and 8 ecological traits, namely (a) nest location (on, above or below ground); (b) life style (ground-dwelling, fossorial, arboreal or a combination); (c) diel activity (diurnal, nocturnal or around the clock); (d) feeding habits (omnivorous, folivorous, granivorous, insectivorous or a combination); (e) occurrence of hibernation or torpor; (f) population density; (g) home range size; (h) dispersal range (the distance between the birth location and the breeding location); (i) social group size; and (j) habitat breadth (according to level 1 IUCN habitats). For example, pre-imaginal development of fleas takes place mainly in a nest of a host, so the location of a nest is associated with temperature and humidity regime which, in turn, affects the survival of pre-imaginal fleas (Krasnov *et al*., 2001). Investment to ‘expensive’ tissue such as brain may compromise immune ability of a host and, thus, facilitate infection by parasites (Bordes *et al*., 2011). The rationale behind the selection of the remaining traits, information sources on traits and details of the calculations of some traits can be found elsewhere (Krasnov *et al*., 2016, 2019*b*, 2023*b*). Prior to the analyses, data on geographic range size (for mammals) were ln-transformed, and then, continuous trait variables for both fleas and mammals were scaled to unit variance and zero mean, whereas nominal trait variables were converted to dummy variables using the function ‘dummy’ in the package ‘BAT’ (Cardoso *et al*., 2023), implemented in the R Statistical Environment (R Core Team, 2023).

### Environmental variables

Data on the latitudes and longitudes of the regions’ centres and on the region-specific values of environmental variables were taken from Krasnov *et al*. (2023*a*). In brief, regional environment was described using (a) the seasonal amount of green vegetation calculated as Normalized Difference Vegetation Indices (NVDI), (b) the mean, maximum and minimum air temperatures and (c) the seasonal precipitation. These data were averaged across 30 arc-second grids separately for each region. Sources of data on the latitudes and longitudes of the regions’ centres, as well on environmental variables, can be found elsewhere (Krasnov *et al*., 2023*a*). Then, each category of environmental variables for each realm was subjected to principal component analyses, and the original values were subsequently substituted with the scores of the first principal components. The resulting 3 composite environmental variables were a vegetation variable (reflecting the amount of green vegetation), an air temperature variable and a precipitation variable. These composite variables reflected an increase in the respective original variables (amount of green vegetation, air temperature and precipitation) and explained from 72 to 97% of the variation in the environmental factors (see details in Krasnov *et al*., 2023*a*).

### Data analyses: functional alpha diversities

The functional richness, functional divergence and functional regularity of both fleas and hosts were calculated using the R package ‘BAT’. First, for each realm and separately for fleas and hosts, we constructed 2 matrices, namely a matrix of species distribution (D-matrix; regions × species) and a matrix of species traits (T-matrix; species × traits). Then, we constructed a neighbour-joining tree for each regional flea or host T-matrix as recommended by Cardoso *et al*. (2022) using the function ‘tree.build’ of the R package ‘BAT’ and Gower's distance. The latter allows constructing a dissimilarity matrix from data composed of continuous, categorical, dichotomous and nominal variables (Gower, 1971). The resulting functional trees and D-matrices were then used to calculate functional richness, functional divergence and functional regularity for fleas and hosts in each realm using the functions ‘alpha’, ‘dispersion’ and ‘evenness’, respectively, of the ‘BAT’ package.

Treating the values of functional diversity components, in different regions within a realm, as independent observations could have introduced a bias in the analysis because multiple flea and host species occurred in more than 1 region. To control for the effects of the same flea and host species in several regions, we analysed the relationships between each component of flea functional diversity and the respective component of host functional diversity, as well as environmental variables, using a modified version of phylogenetic generalized least squares (PGLS; Martins and Hansen, 1997; Pagel, 1997, 1999; Rohlf, 2001). Classical PGLS is applied in comparative analyses to account for interspecific autocorrelation due to phylogeny and, thus, controls for non-independence of data points (i.e. species related to each other *via* phylogeny). Here, we controlled for non-independence of regional data within a realm by substituting a phylogenetic tree with a realm-specific dendrogram of regions based on similarity in species composition of both fleas and hosts. For this, we (a) combined flea and host D-matrices for each realm; (b) constructed, from this matrix, a matrix of dissimilarity on flea and host species composition using the function ‘vegdist’ of the R package ‘vegan’ (Oksanen *et al*., 2022) with the option method = ‘bray’; (c) built a cluster dendrogram using the function ‘hclust’ of the R package ‘stats’ (R Core Team, 2023) with the option method = ‘complete’; and (d) transformed the resulting dendrogram into a pseudo-phylogenetic tree using the function ‘as.phylo’ of the R package ‘ape’ (Paradis and Schliep, 2019).

Then, we applied these modified PGLSs to test the relationships between each component of flea functional diversity (response variables) and the respective component of host functional diversity and the 3 composite environmental variables (explanatory variables) separately for each realm. We ran each model and applied forward stepwise model selection using the function ‘phylostep’, implemented in the R package ‘phylolm’ (Ho and Ane, 2014). For each model, we tested for residual spatial autocorrelation (Kühn and Dormann, 2012) using Moran's *I* metric with the R package ‘ape’. No residual spatial autocorrelation was detected in any model (Moran's *I*, *P* > 0.07 for all).

### Data analyses: functional beta diversities

Functional beta diversity essentially represented functional dissimilarity between regions and, thus, was analogous to traditional dissimilarity metrics in which species are replaced by functional units. To investigate the relationships between the functional beta diversities of fleas and hosts, as well as to test for the effects of environment and geographic distance between regions, we applied a modified version of phylogenetic generalized dissimilarity modelling (phyloGDM) which, in turn, is an extension of generalized dissimilarity modelling (GDM) (Ferrier *et al*., 2007; Mokany *et al*., 2022). In general, GDM tests the relationships between the species turnover of one taxon with that of another taxon and/or species turnover and environmental dissimilarity. An advantage of GDM is that it controls for 2 main problems associated with the linear analyses of species turnover or between-site dissimilarity, namely (a) variation of any dissimilarity metric from 0 to 1 only, and (b) a non-constant rate of species turnover along a gradient. In particular, the GDM transforms each predictor using an iterative maximum-likelihood estimation and I-splines, thus accounting for the curvilinear fashion of the turnover rate variation along a gradient (Ferrier *et al*., 2007; Fitzpatrick *et al*., 2022). The maximum height of each I-spline corresponds to the total amount of turnover associated with a given gradient, holding all other predictors constant. In other words, each I-spline is a partial regression fit that reflects the importance of each predictor's effect on species turnover, whereas the slope of an I-spline demonstrates not only the rate of the turnover, but also the variation of this rate along a gradient. Moreover, the GDM can incorporate various biotic and abiotic predictors into a single model. In phyloGDM, species are replaced with phylogenetic lineages and, thus, spatial patterns of phylogenetic turnover (Ferrier *et al*., 2007; Nipperess *et al*., 2010; Rosauer *et al*., 2013; Pavoine, 2016). We modified the phyloGDM by using a functional rather than a phylogenetic tree, thus replacing the species of the original GDM with functional units.

We used flea and host D-matrices and functional flea and host trees for each realm. From these matrices and trees, we constructed flea and host functional dissimilarity matrices using the function ‘evodiss_family’ of the R package ‘adiv’ (Pavoine, 2020), using coefficient S12 of Gower and Legendre (1986) based on Ochiai (1957) (because this coefficient is calculated for incidence rather than abundance data). Functional GDMs were carried out for each realm using the R package ‘gdm’ (Fitzpatrick *et al*., 2022) to test for the relationships between (a) flea functional dissimilarity ( = beta diversity = turnover of functional units) and (b) host functional dissimilarity, environmental dissimilarity and geographic distances. Model and predictor significance testing were estimated using matrix permutation with the function ‘gdm.varImp’ of the package ‘gdm’.

## Results

### Functional alpha diversity

**Figure 1.**
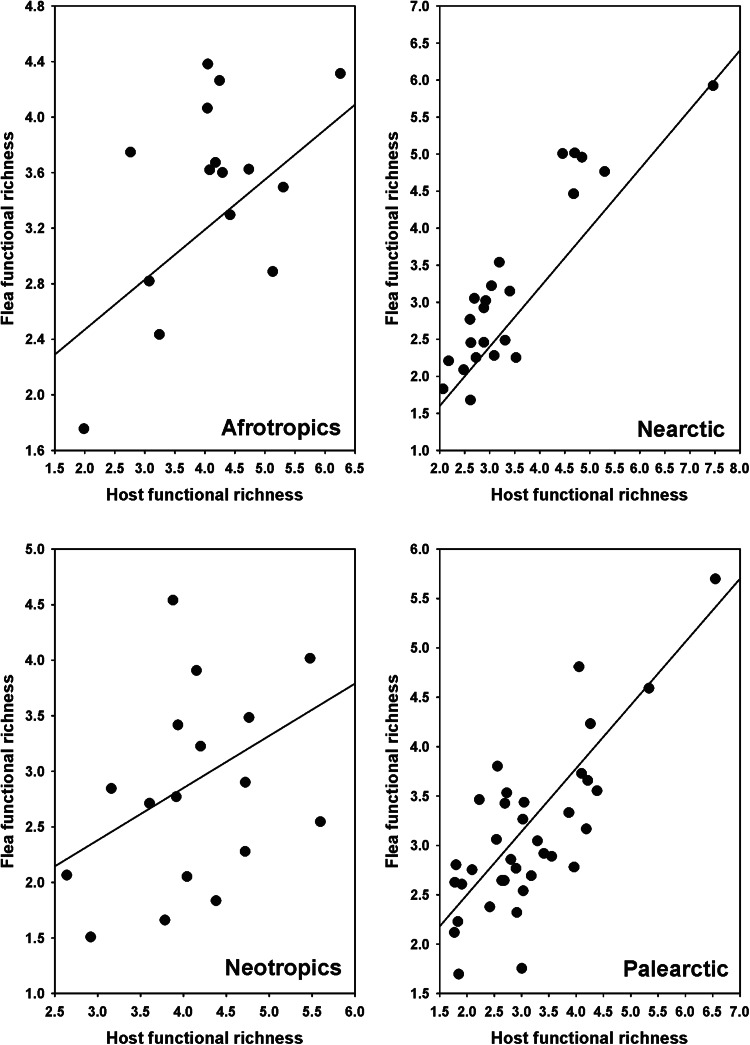
Relationships between flea functional richness and host functional richness across regions in 4 biogeographic realms. Coefficients of the regression lines are from phylogenetic generalized least squares.

**Figure 2.**
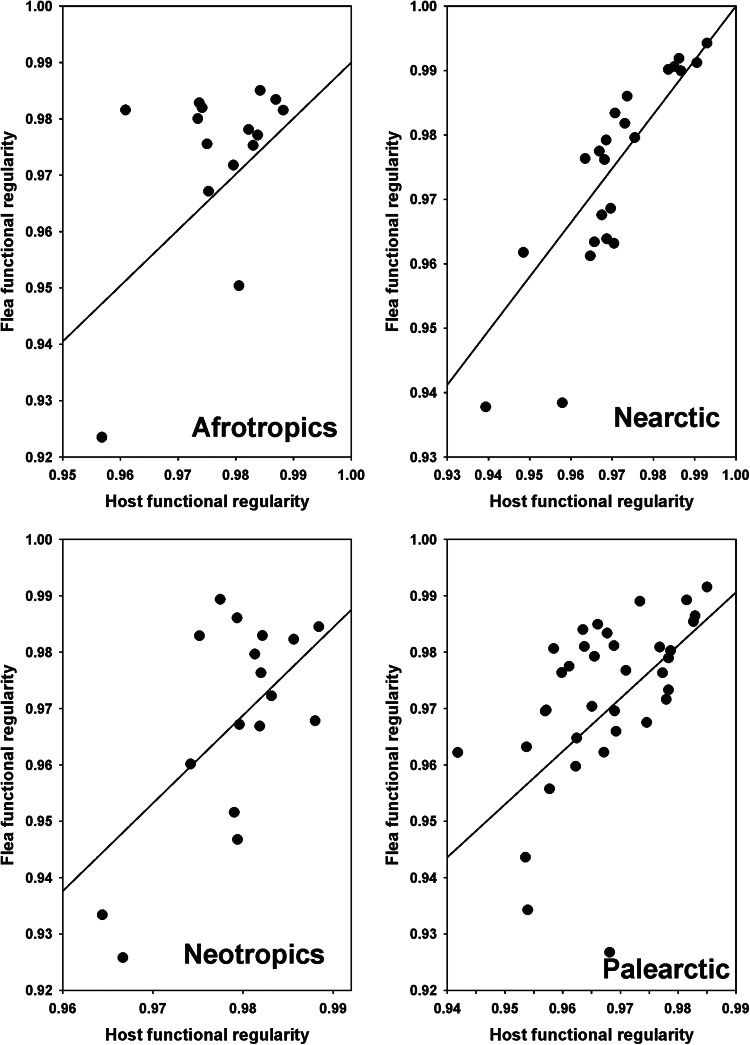
Relationships between flea functional regularity and host functional regularity across regions in 4 biogeographic realms. Coefficients of the regression lines are from phylogenetic generalized least squares.

**Figure 3.**
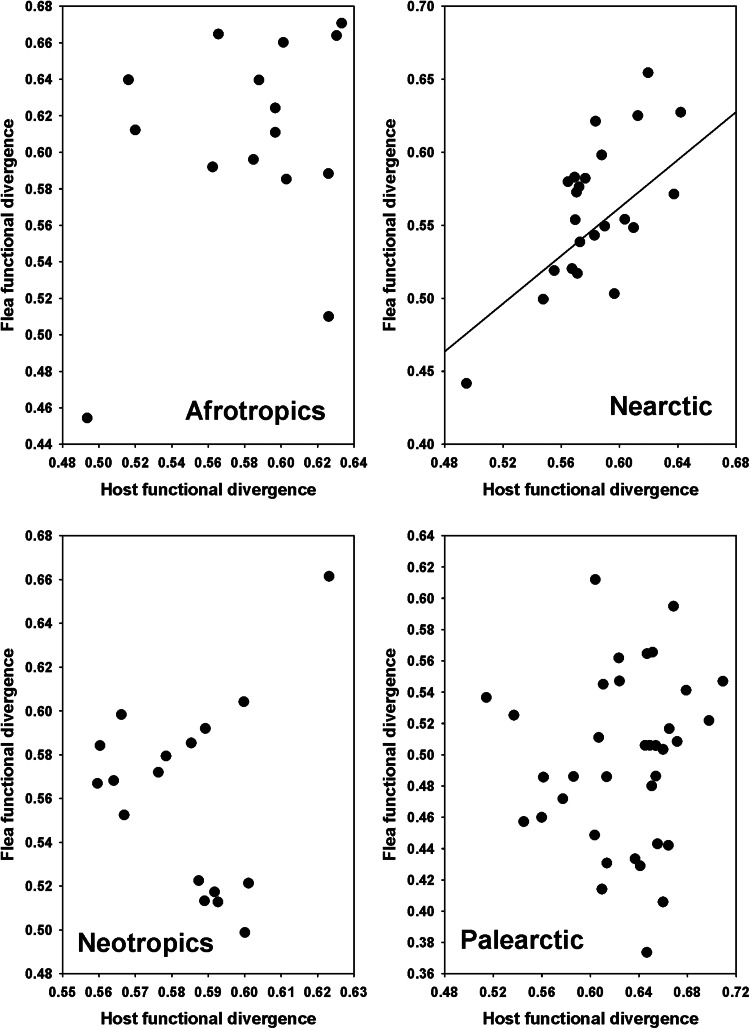
Relationships between flea functional divergence and host functional divergence across regions in 4 biogeographic realms. Coefficients of the regression lines for the Nearctic are from phylogenetic generalized least squares.

The results of the PGLS of the relationships between components of flea functional diversity and the respective components of host functional diversity and environmental variables are presented in Table 1. In all realms, flea functional richness increased with an increase in host functional richness (Fig. 1). The same was the case for functional regularity (Fig. 2). On the contrary, flea functional divergence correlated (positively) with that of hosts in the Nearctic only (Fig. 3). Regarding the effects of environment, flea functional richness was not affected by any environmental factor, whereas their functional divergence correlated positively with air temperature in the Nearctic and precipitation in the Palearctic (Fig. S1, Appendix 1, Supplementary Material). No effect of environment on this component of flea functional diversity was found in the remaining realms. An environmental effect on flea functional regularity was detected in the Nearctic only (increase with a decrease in the amount of green vegetation) (Fig. S1, Appendix 1, Supplementary Material).

**Table 1.** Summary of stepwise phylogenetic generalized least squares (PGLS) of the relationships between components of flea functional alpha diversity (richness, divergence and regularity) and the respective components of host functional alpha diversity and environmental variables (Veg, T, P) in 4 biogeographic realms

Realm	Component of functional diversity	Equation	*R^2^*
Afrotropics	FFRi	1.75* + 0.36HFRi*	0.31
	FFD	–	
	FFRe	0.99HFRe*	0.29
Nearctic	FFRi	0.80HFRi***	0.79
	FFD	0.58HFD* + 0.01 T*	0.56
	FFRe	0.78HFRe***–0.004Veg**	0.70
Neotropics	FFRi	0.97* + 0.47HFRi**	0.50
	FFD	–	
	FFRe	1.66HFRe**	0.49
Palearctic	FFRi	1.21*** + 0.64HFRi***	0.65
	FFD	0.02P*	0.15
	FFRe	0.92HFRe***	0.34

FFRi and HFRi: functional richness of fleas and hosts, respectively; FFD and HFD: functional divergence of fleas and hosts, respectively; FFRe and HFRe: functional regularity of fleas and hosts, respectively; Veg, T and P: composite environmental variables reflecting the amount of green vegetation, air temperature and precipitation, respectively.

Only significant predictors are shown. *P < 0.05, **P < 0.01, ***P < 0.001.

### Functional beta diversity

**Figure 4.**
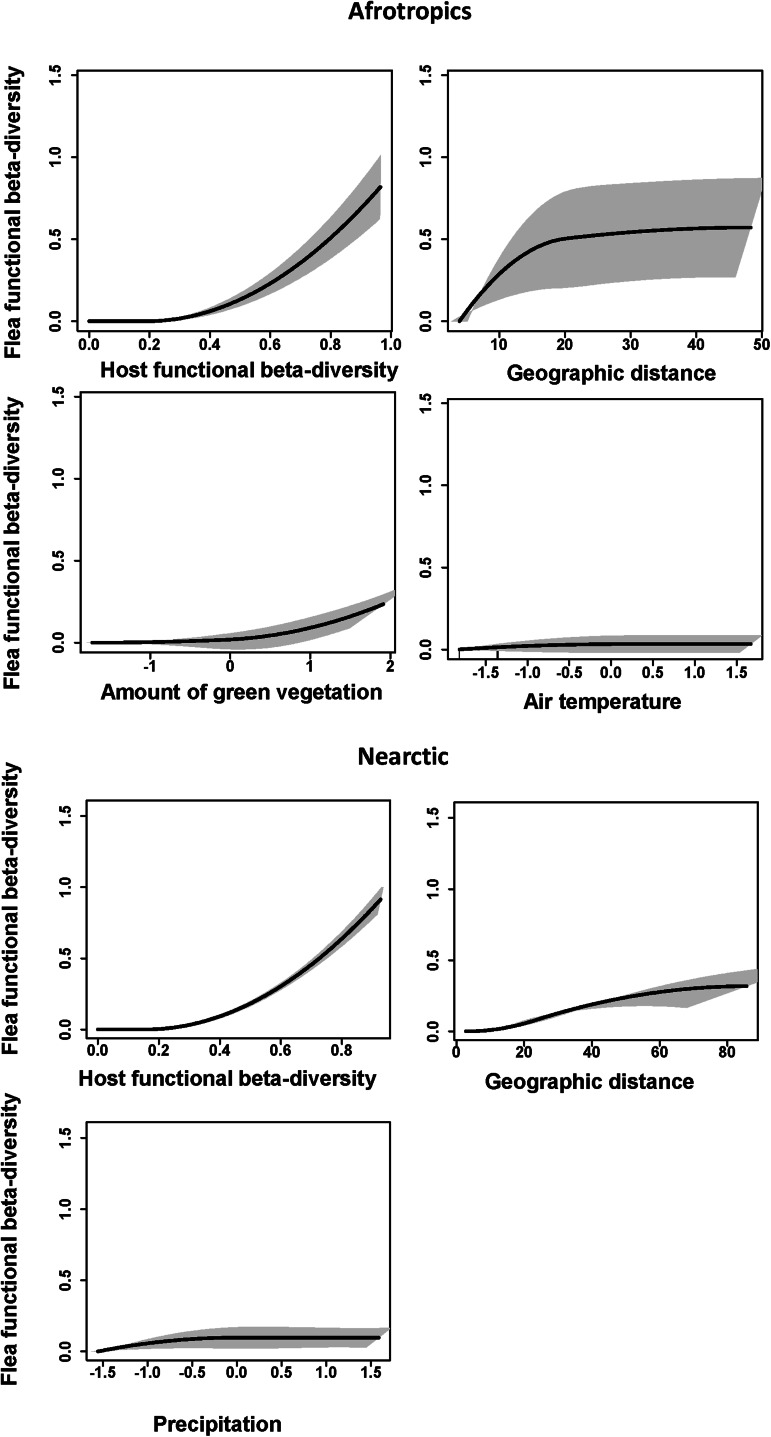
Generalized dissimilarity model-fitted I-splines and 95% confidence intervals (partial regression fits) of host functional turnover, environmental variables and geographic distance as predictors of flea functional turnover in the Afrotropics and the Nearctic. The steeper slope of an I-spline shows a greater rate of turnover at a given gradient part.

**Figure 5.**
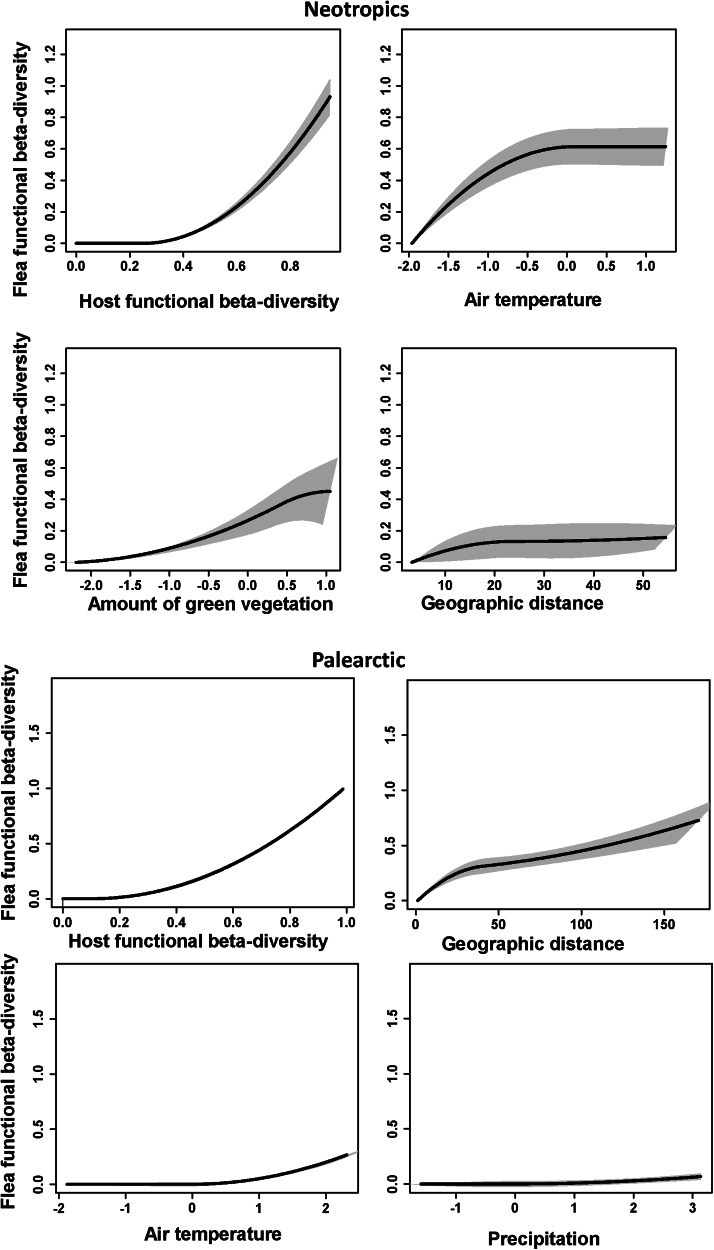
Generalized dissimilarity model-fitted I-splines (partial regression fits) and 95% confidence intervals of host functional turnover, environmental variables and geographic distance as predictors of flea functional turnover in the Neotropics and the Palearctic. The steeper slope of an I-spline shows a greater rate of turnover at a given gradient part.

The GDM models for the effects of host functional turnover, environmental gradients and geographic distance on flea functional turnover explained about 75–80% of deviance in all realms (Table 2). Host functional turnover was invariantly the best predictor of flea functional turnover (Tables 2–3) with the rate of the latter being higher at the higher values of the former (Figs 4–5). On the contrary, the effects of environmental variables on flea functional turnover were much weaker, with the roles of different environmental factors being different in different realms. In particular, the air temperature gradient appeared to be a strong predictor of flea functional turnover in the Neotropics, whereas the effect of this factor was much weaker in the Palearctic and mostly lacking in the Afrotropics and the Nearctic (Tables 2–3). In all realms, the functional dissimilarity of regional flea assemblages did not vary, or barely varied, along the precipitation gradient (Tables 2–3, Figs 4–5), whereas some effect of the vegetation gradient was found in the realms of the Southern but not the Northern Hemisphere. Between-region geographic distance was the second-best (after host functional turnover) predictor of flea functional turnover in the Afrotropics, the Nearctic and the Palearctic (Tables 2–3). However, this effect in the Nearctic was rather weak, whereas in the Neotropics, it was much less important than the effects of the air temperature and vegetation gradients (Tables 2–3).

**Table 2.** Flea functional beta diversity as explained by host functional beta diversity (HFBD), environmental variables (Veg, T, P) and geographic distance (GD) between regions in 4 biogeographic realms

Realm	%Deviance explained	Predictor	I-spline 1	I-spline 2	I-spline 3	Σ_I-splines_
Afrotropics	81.10	HFBD	0.00	0.00	1.09	1.09
		Veg	0.00	0.04	0.20	0.24
		T	0.03	0.00	0.00	0.03
		P	0.00	0.00	0.00	0.00
		GD	0.46	0.11	0.00	0.57
Nearctic	85.76	HFBD	0.00	0.00	1.49	1.49
		Veg	0.00	0.00	0.00	0.00
		T	0.00	0.00	0.00	0.00
		P	0.10	0.00	0.00	0.10
		GD	0.00	0.32	0.00	0.32
Neotropics	75.18	HFBD	0.00	0.00	1.23	1.23
		Veg	0.03	0.42	0.00	0.45
		T	0.61	0.00	0.00	0.61
		P	0.00	0.00	0.10	0.10
		GD	0.13	0.00	0.03	0.16
Palearctic	76.38	HFBD	0.00	0.00	1.99	1.99
		Veg	0.00	0.00	0.00	0.00
		T	0.00	0.00	0.27	0.27
		P	0.00	0.00	0.06	0.06
		GD	0.28	0.14	0.31	0.73

Veg, T and P: composite environmental variables reflecting the amount of green vegetation, air temperature and precipitation, respectively; I-splines 1, 2 and 3: coefficients of the first, second or third I-spline, respectively; Σ_I-splines_: sum of 3 I-splines (demonstrates the amplitude of an I-spline). An I-spline is a partial regression fit that reflects the importance of each predictor's effect on functional turnover, whereas the slope of an I-spline demonstrates the rate of functional turnover as well as the variation of this rate along a gradient. The maximum height of each I-spline corresponds to the total amount of turnover associated with a given gradient while holding all other predictors constant.

**Table 3.** Relative importance of host functional beta diversity (HFBD), environmental variables (Veg, T, P) and geographic distance (GD) for flea functional beta diversity calculated by generalized dissimilarity modelling

Realm	HFBD	Veg	T	P	GD
Afrotropics	14.17	1.46	1.17	0.00	5.75
Nearctic	38.65	0.00	0.00	0.50	2.10
Neotropics	14.05	2.65	7.64	0.38	0.34
Palearctic	56.67	0.10	0.84	0.11	3.89

Importance of a predictor is estimated using matrix permutation and is measured as the per cent decrease in deviance explained between the full model and the deviance explained by a model with the predictor permuted.

Veg, T and P: composite environmental variables reflecting the amount of green vegetation, air temperature and precipitation, respectively.

## Discussion

Our results demonstrated that host functional alpha diversity, in terms of functional richness and regularity, and functional beta diversity are the main drivers of the respective aspects of flea functional diversity. These patterns appeared to be invariant across biogeographic realms, i.e. they did not depend on the identities of either host or fleas. This, however, was not the case for functional divergence. In contrast to the effects of host functional diversity (except divergence), the effects of environmental factors on flea functional alpha and beta diversity differed substantially between realms, suggesting that this between-realm difference can be associated with between-realm variation in flea species composition, with different species responding differently to environmental variation, in terms of their traits.

### Flea and host functional diversities

It is obvious that parasites ultimately depend on their hosts, so they must be able to extract resources from the hosts and to overcome their defence efforts. Consequently, parasites should evolve traits allowing them to successfully obtain resources from hosts, with these traits being determined by the respective host traits. For example, the positive relationships between (a) the head-groove width of chewing lice and the hair-shaft diameter of their gopher host, on the one hand, and (b) body size and head-groove width in lice, on the other hand, have lead Morand *et al*. (2000) to conclude that evolutionary changes in the body size of chewing lice are driven by a relationship between the parasite's head-groove dimension and the diameter of its host's hairs. Fleas with both a genal comb and a pronotal comb have been shown to exploit mainly small-bodied hosts characterized by high metabolic rates (Krasnov *et al*., 2016). The likely reason for this is that combs allow fleas to anchor themselves to the host hair and thus resist dislodgement by host grooming (e.g. Traub, 1985). Consequently, fleas could develop both combs to be able to parasitize (a) smaller hosts that groom harder to decrease the number of parasites per unit body surface (Mooring *et al*., 2000) and (b) hosts investing in a higher metabolic rate as a compensation for costly behavioural defences (Giorgi *et al*., 2001). Fleas with greater jumping abilities (estimated *via* morphological features such as pleural height) and larger geographic ranges were found to exploit bird hosts with smaller social groups, thus increasing the probability of between-host transmission (Tripet *et al*., 2002). Trait-matching can thus explain the generally positive relationships between the functional diversities of parasites and hosts found in this study. However, parasite–host trait-matching does not always occur due to the existence of so-called exploitation barriers (e.g. Santamarıá and Rodríuez-Gironé, 2007), through, for example, the development of stronger anti-parasitic defences (Fellowes *et al*., 1999; Nuismer and Thompson, 2006). In antagonistic interactions, barriers are naturally evolved mechanisms for blocking exploitation such as development of traits that prevent exploitation (Goodman and Ewald, 2021). For instance, coevolutionary alternation describes cyclic evolutionary fluctuations in predator/parasite preferences driven by evolutionary shifts in prey/host defences and vice versa (Nuismer and Thompson, 2006). Trait-matching (= trait complementarity) can be translated into structural patterns of interaction networks (McQuaid and Britton, 2013). Theoretically, trait-matching can arise not only due to parasite adaptations to host traits, but also from the effects of parasite on host trait composition (Frainer *et al*., 2018), altering, for example, host mobility, habitat preferences or body size (e.g. Miura *et al*., 2006). However, this is highly unlikely for fleas because they are not known to be able to manipulate the physiology, morphology or behaviour of their host at the scale of the host species (although they cause these changes in individual hosts; e.g. Khokhlova *et al*., 2002).

Another, not necessarily alternative, reason for the positive relationships between flea and host functional diversities may result from the tight relationship between flea and host phylogenetic diversities (Krasnov *et al*., 2023*a*; but see Yaxley *et al*., 2023) since many traits of both fleas and hosts are phylogenetically conserved. In fleas, phylogenetically conserved traits include, for example, body size (Surkova *et al*., 2018), latitudinal position and size of geographic range (Krasnov *et al*., 2018*a*, 2018*b*) and characteristic abundance (Krasnov *et al*., 2011), whereas in hosts, such traits include, among others, body size (Capellini *et al*., 2010), relative brain mass (Antoł and Kozłowski, 2020) and dispersal distance (Whitmee and Orme, 2012). However, the relationships between functional and phylogenetic diversities can be scale-dependent. For example, in birds, they show substantial variation across latitudes (Yaxley *et al*., 2023).

As mentioned above, geographically invariant positive relationships between flea and host functional diversities were found for functional richness, functional regularity and functional beta diversity but not for functional divergence. In other words, the community-wise amount of flea functions (functional richness) and the degree of variability of these functions (functional regularity) correlated with those of hosts independently of species composition and evolutionary history of flea and host communities as well as their geographic patterns of dispersal. Geographic invariance of the relationships between flea and host functional beta diversities suggested that functional turnover ( = dissimilarity) of fleas followed functional turnover ( = dissimilarity) of hosts whatever species compositions of fleas and hosts are. For the functional divergence, positive relationships were detected in the Nearctic only. Functional divergence represents the answer to the question: how different are species in their functional traits? One of the reasons for this may be the history of the Nearctic flea fauna as compared with those in other realms. The Nearctic fleas are heavily represented by the members of the youngest family Ceratophyllidae (Medvedev, 2005). Furthermore, flea historical dispersal between the Palearctic and Nearctic at the pre-glaciation time *via* the Bering Land Bridge is thought to have occurred primarily eastward, resulting in the recent flea clades being represented mainly in North America (Medvedev, 2005; Krasnov *et al*., 2015*a*). These lines of evidence suggest a shorter history of flea–host associations in the Nearctic. The longer histories of flea–host associations in the remaining realms could lead to some kind of homogenization of flea traits in relation to host traits, whereas this probably was not the case for the Nearctic due to the shorter time of adaptation to hosts.

### Flea functional diversity and environment

Environmental factors had much weaker, albeit not negligible, effects on flea functional dissimilarity than host functional turnover, indicating variation in some flea traits along environmental gradients. This may be the result of environmental filtering of flea assemblages when the environment constrains a community composition only to species possessing certain adaptive traits that are necessary for persistence in that environment (Cavender-Bares *et al*., 2004; Ingram and Shurin, 2009). In particular, environmental filtering has been shown to be a mechanism of compound regional flea community assembly in the Palearctic (Krasnov *et al*., 2015*b*). In fact, fleas in the regions with lower air temperatures were characterized by larger body size and lower host specificity, whereas fleas from the regions with higher air temperature appeared to be smaller and their host specificity was relatively high (Krasnov *et al*., 2008, 2015*b*, 2023*c*). Nevertheless, the environmental effects on flea functional alpha-diversity were found in both Holarctic realms but not in the realms of the Southern Hemisphere, whereas environmental predictors of flea functional beta-diversity differed between realms. This could be because the variation in environmental factors differs between realms. For example, climatic conditions in the Holarctic range from hot deserts to cold tundra *via* the temperate zone, whereas climatic gradients in the Southern Hemisphere realms seem to be shorter, especially given that no flea samplings were carried out in the southernmost parts of South America. Another reason might be differences in trait distribution along environmental gradients in different flea species inhabiting different realms, likely due to between-realm differences in flea evolutionary history, as well as historical events such as glaciation. We recognize that these explanations are highly speculative and warrant further investigation. Interestingly, environmental factors affecting flea beta diversity within a realm differed between functional (this study) and phylogenetic (Krasnov *et al*., 2023*a*) beta diversities. This supports the recent ideas that (a) phylogenetic diversity might be an unreliable surrogate of functional diversity and (b) the relationship between phylogenetic diversity and functional diversity is context dependent (Yaxley *et al*., 2023).

Finally, geographic distance appeared to be the second-best predictor of flea functional beta diversity, especially in the realms of the Southern Hemisphere. This can be considered as a manifestation of the widely recognized ecological pattern of distance decay of similarity (Nekola and White, 1999). Distance decay of compositional and phylogenetic similarity has earlier been shown for fleas in some but not other biogeographic realms (Krasnov *et al*., 2012, 2023*a*), suggesting that it may not be universal, not only in terms of compositional or phylogenetic similarity (Vinarski *et al*., 2007; Pérez-del-Olmo *et al*., 2009; Maestri *et al*., 2017), but also in terms of functional similarity.

In conclusion, flea functional alpha and beta diversities are mostly driven by host functional alpha and beta diversities, with these patterns being geographically invariant. In contrast, environmental effects on flea functional alpha and beta diversities vary geographically. In addition, environmental effects on flea functional alpha diversity differ between its components.

## Supporting information

Krasnov et al. supplementary material Krasnov et al. supplementary material

## Data Availability

Data on flea and host species composition in the Afrotropics, the Neotropics, the Nearctic and the Palearctic are deposited in the Mendeley Data Repository: 10.17632/dzyvrp7kfh.2 (Krasnov, 2023).

## Supplementary material

The supplementary material for this article can be found at https://doi.org/10.1017/S0031182024000283.
