# Supplementary material for: Relationships between functional alpha and beta diversities of flea parasites and their small mammalian hosts
Source: Parasitology. 2024 Mar 4;151(4):449–60. doi: 10.1017/S0031182024000283 (PMC11043902; doi:10.1017/S0031182024000283)

**Appendix 1.**

**Appendix 1. Supplementary figures**

**Fig. S1.** Relationships between (A) flea functional divergence and air temperature in the Nearctic; (B) flea functional regularity and the amount of green vegetation in the Nearctic; and (C) flea functional divergence and vegetation in the Palearctic. Coefficients of the regressions lines are from phylogenetic generalised least squares.


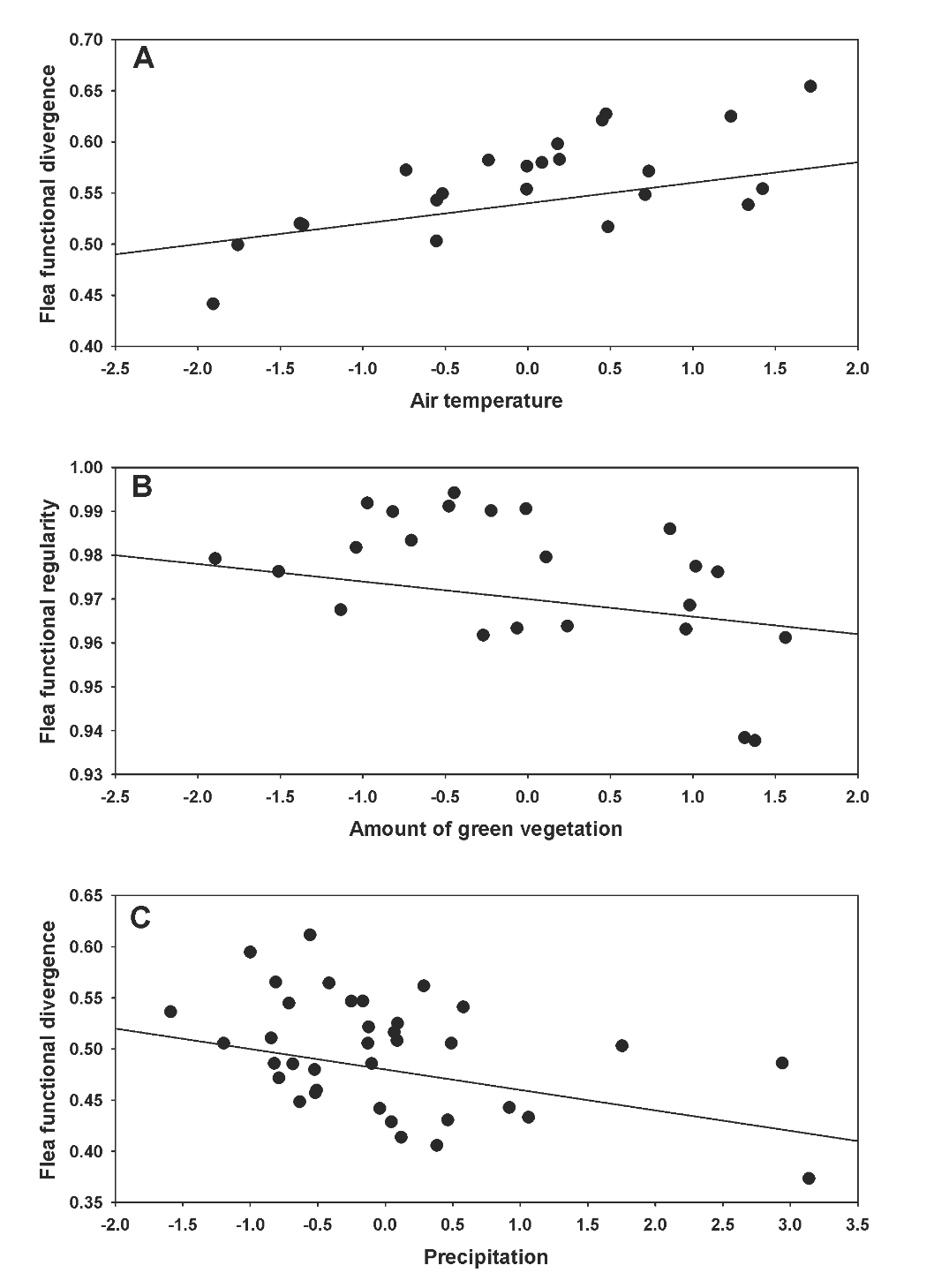

Supplement: Krasnov et al. supplementary material [file S0031182024000283sup001.docx]
